# Are microtubules tension sensors?

**DOI:** 10.1038/s41467-019-10207-y

**Published:** 2019-05-29

**Authors:** Olivier Hamant, Daisuke Inoue, David Bouchez, Jacques Dumais, Eric Mjolsness

**Affiliations:** 1 Laboratoire de Reproduction et Développement des Plantes, Université de Lyon, UCB Lyon 1, ENS de Lyon, INRA, CNRS, 46 Allée d’Italie, 69364 Lyon Cedex 07, France; 2 Cell and Plant Physiology Laboratory, CytoMorpho Lab, CEA, Biosciences and Biotechnology Institute of Grenoble, 38054 Grenoble, France; 3 Institut Jean-Pierre Bourgin, INRA, AgroParisTech, CNRS, Université Paris-Saclay, 78000 Versailles, France; 4 Facultad de Ingeniería y Ciencias, Universidad Adolfo Ibáñez, Viña del Mar, Region V Chile; 5 Departments of Computer Science and Mathematics, University of California, Irvine, CA 92697-3435 USA

**Keywords:** Biophysics, Microtubules, Plant cytoskeleton, Shoot apical meristem

## Abstract

Mechanical signals play many roles in cell and developmental biology. Several mechanotransduction pathways have been uncovered, but the mechanisms identified so far only address the perception of stress intensity. Mechanical stresses are tensorial in nature, and thus provide dual mechanical information: stress magnitude and direction. Here we propose a parsimonious mechanism for the perception of the principal stress direction. In vitro experiments show that microtubules are stabilized under tension. Based on these results, we explore the possibility that such microtubule stabilization operates in vivo, most notably in plant cells where turgor-driven tensile stresses exceed greatly those observed in animal cells.

## Abstract (web summary)

Cellular mechanical stress is a key determinant of cell shape and function, but how the cell senses stress direction is unclear. In this Perspective the authors propose that microtubules autonomously sense stress directions in plant cells, where tensile stresses are higher than in animal cells.

## Introduction

Mechanical forces are increasingly viewed as instructive signals for many cell biology processes, such as cell polarity^1^, division^2^, and fate^3^. Mechanical forces also play important roles in developmental biology. For instance, tissue folding during gastrulation in Drosophila^4^ or during organogenesis in plants^5^ involves a response of the cytoskeleton to mechanical forces. Similarly, the mechanical conflicts associated with differential growth in organs constrain their final shape, both in animals^6,7^ and plants^8^. Several mechanotransduction pathways have been identified^9^, yet there is no clear mechanism for sensing stress direction so far. Typically, membrane tension is thought to open mechanosensitive channels, through membrane thinning^10^. However, the plasma membrane is fluid, and thus can only be under isotropic tension, like a soap film. The transmission of stress direction through the membrane requires a coupling with an elastic solid, such as the cell wall, the extracellular matrix, or the cortical cytoskeleton. Because cytoskeletal proteins are structurally and dynamically directional, they may be inherently more sensitive to the directionality of mechanical cues. Interestingly, the cytoskeleton has been proposed to respond directly to mechanical stimuli, making this structure not only a good substrate for the transmission of mechanical information but also a potential contributor to the transduction of stimuli. For instance, in single cells, tension modifies formin conformation, from an inhibitory to a permissive one, thereby promoting actin polymerization^11^, whereas compression can promote actin branching, thereby affecting the contractile behavior of the cell cortex^12^.

More generally, to sense direction, one needs an anisotropic probe. Microtubules may be particularly well-suited for this function not only because their shape makes them typically anisotropic, but also because these molecules are remarkably stiff. In fact, the 25 nm-wide microtubules are three orders of magnitude stiffer than actin^13^, endowing them with a high persistence length and the ability to maintain their shape and anisotropy. Furthermore, and maybe more importantly, the bending stiffness of microtubules allows them to maintain a given direction over the whole cell or at least a large part of it. Thus, the mechanical properties of microtubules, together with their extended shape, make them well-suited to perceive cell-scale mechanical signals.

Here, in the spirit of a perspective, we explore the possibility that individual microtubules are able to sense their own longitudinal tensile status, and to align spontaneously with the direction of maximal tensile stress (mathematically, the principal axis of the stress tensor, in living cells and tissues; key terms are defined in Box 1).

Plants are ideal systems to study this question for two main reasons. First, turgor pressure in plant cells routinely exceeds 0.5 MPa, which builds up high tensile stresses at the cell cortex, where a dense population of microtubules (so-called cortical microtubules, CMTs) self-organize in a confined 2D space. Second, there is overwhelming evidence that plant cortical microtubules respond quickly to changes in the stress in plant cells^5,14,15^. Therefore, by virtue of their extended nature, their high persistence length, their position at the cell cortex, and their rapid response to changes in wall stresses, microtubules are the best candidate as the cellular structure able to sense the direction of stress.

Note that because of the presence of stiff cell walls, the only origin of mechanical stress in plant cells is turgor pressure. Spindle microtubules are known to generate a pushing or pulling force when they grow or shrink, respectively. However, such forces are small: the addition of 13 dimers (i.e., a full 8 nm tall ring of tubulin dimers) is thought to generate a force of ~50 pN^16^. Given that the stiffness of cell walls is in the MPa range, such forces are negligible. In fact, plant cells do not change their shape for several hours after microtubules have been depolymerized^17,18^.

**Fig. 1:**
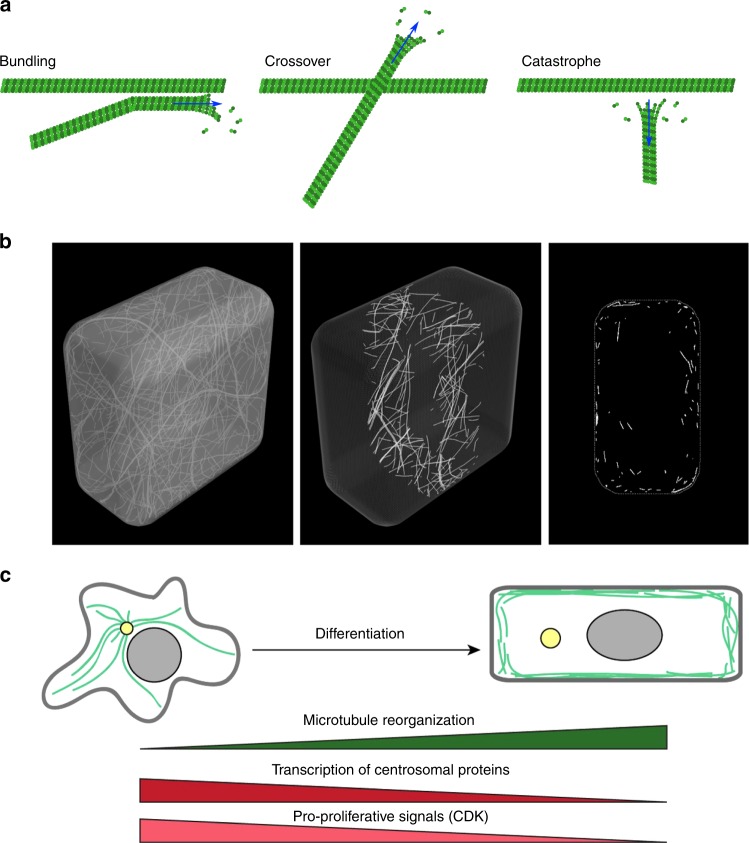
Microtubule self-organization properties lead to their cortical localization by default. **a** Dynamic instability and self-organizing properties of microtubules. Bundling occurs for collision angles inferior to 40°; for larger angles, induced catastrophes or crossover occur. **b** Microtubules are cortical by default in silico (adapted from ref. ^31^). **c** Upon centrosome disorganization, microtubules can become cortical in differentiated animal cells (adapted from ref. ^32^)

How could microtubules align with the direction of maximal tension? Does this response require a specific mechanotransduction pathway? Plant microtubules are comparable to microtubules found in animal systems^19,20^, although they exhibit increased dynamics: using purified plant tubulin assembled in vitro, catastrophe was found to be more frequent than in animals and the shrinking rate was almost 10 times higher in plants than in animals (195 μm/min for plant microtubules vs. 21 μm/min for animal microtubules)^21^. γ-TuRCs are located both at the nuclear envelope and the plasma membrane, yet, the plasma membrane is generally thought to be a dominant site for microtubule nucleation, at least during interphase^22,23^. The alignment of CMTs with tension likely involves self-organization processes (Fig. 1a). Beyond nucleation, CMTs have indeed been shown to form organized arrays spontaneously, through the combination of their (de)polymerization, bundling, and severing^24,25^. Microtubules are dynamic at both plus and minus ends, and the associated bias in dynamic instability results in treadmilling events^26^. Short treadmilling microtubules (0.5–2 μm) represent ca. 90% of the treadmilling microtubules and have a major contribution to the final CMT organization^27^. Although the microtubule lattice is more dynamic than initially anticipated^28^, it is now well established that the microtubule ends play a major role in microtubule stability. The SPIRAL2 protein for instance was recently shown to bind and stabilize microtubule minus ends, indirectly impacting the severing rate^23,29^. More directly, tumor overexpressed gene (TOG)-domain containing proteins such as XMAP215 incorporate free tubulin at the microtubule plus ends and thus catalyze microtubule polymerization.

Although the exact contribution of each process to CMT alignment with tension remains to be investigated, katanin-dependent MT severing has been shown to promote CMT alignment with tensile stress^30.^ This raises the question of the number and identity of players needed for microtubules to sense tension. In this perspective, we speculate about the most parsimonious hypothesis: could individual microtubules align with rapid changes in maximal tension direction on their own without the help of other factors?

Box 1 Definitions

- Stress (*σ*): a force per unit area upon which the force is acting (measured in Pa). Stress is also the mathematical product of stiffness and strain (*σ* = *Eε*).
- Strain (*ε*): a normalized and unitless measure of deformation (in 1D for an object of length *l*, ε = (*l−l*_*0*_)/*l*_*0*_).
- Stretch (*η*): a unitless measure of deformation (in 1D for an object of length *l*, *η* = *l*/*l*_*0*_).
- Stiffness (*E*): a measure of the rigidity of the material. *E* corresponds to the elastic modulus of the material (in Pa). *E* = *σ/ε*.
- Persistence length: a measure of the stiffness of a linear polymer, as the length over which correlations in the direction of the tangent are lost.

## Contribution of cell geometry to microtubule organization

Since tension is borne by the  cell walls, the localization of CMTs puts them in the ideal location to sense such cortical cues. Interestingly, the cortical localization of CMTs was recently proposed to emerge from their intrinsic stiffness. When growing geometrically stiff microtubules in a closed 3D space and allowing them to self-organize in arrays in silico, they tend to populate the cortex of the cell: when microtubules reach the cell membrane, they may grow in the plane of the membrane without returning to the cell volume; the cell surface thus acts as a microtubule sink in a positive feedback loop^31^ (Fig. 1b). Although this obviously does not exclude anchoring molecules (Box 2), microtubule localization already exhibits a bias that makes them more prone to sense cortical signals on their own. Interestingly, when animal cells differentiate, they tend to loose their centrosome, and this is accompanied by an increase in the cortical localization of microtubules^32^ (Fig. 1c), also consistent with the model’s prediction. The increased density of nucleating proteins, such as GCP proteins and pre-existing microtubules^22,33^, would further confine microtubules to the cell cortex.

**Fig. 2:**
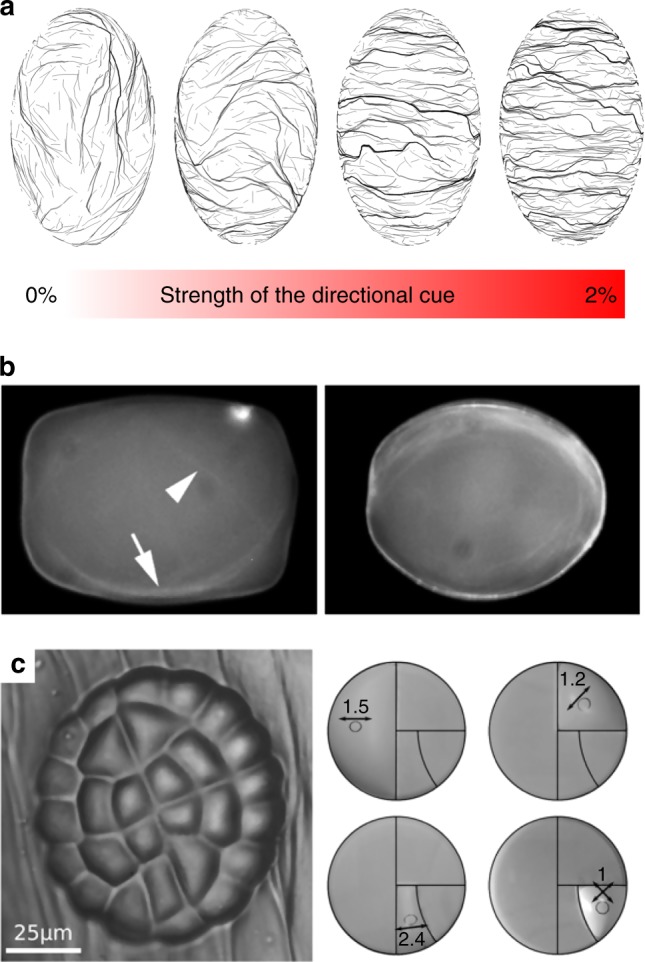
Microtubules are sensitive to cell geometry. **a** In silico, microtubule-bending stiffness weakly influences their final alignment towards the longitudinal axis of the cell; cell geometry also prescribes maximal tension along the transverse direction of the cell, which may in turn counteract the effect of confinement on the final microtubule configuration (adapted from ref. ^31^). **b** In vitro, microtubules can align with the longitudinal axis of confined spaces. In the present study, most (71%), rhodamine-labeled microtubules aligned along the longitudinal axis of confined space in vitro after 1 h of incubation at room temperature (adapted from ref. ^34^). **c**. Left division pattern in the glandular trichome of *Dionaea muscipula;* right: predicted maximal tension directions in the membranes (deformed circles) matching division planes (adapted from ref. ^89^)

In the above-mentioned 3D model of MT self-organization, and confirming previous work, CMTs were shown to be sensitive to cell geometry and to align with the long axis of the cell^31^ (Fig. 2a). At the individual microtubule level, high persistence length would make them avoid the curvy parts of the cell. As they self-organize into complex arrays, such bias would be sufficient to generate microtubule networks aligning along the straightest parts of the cell, i.e., along the longest wall of a plant cell. Several in vitro assays in cell-sized microchambers also reproduced microtubule orientation along the longitudinal axis of such confined space (Fig. 2b^34,35^). Detailed analysis of microtubule behavior in *clasp* mutant further supports this view: CLIP170-associated protein (CLASP) has indeed been proposed to help microtubules continue to polymerize as they bend around sharp cell edges; in the *clasp* mutant, increased rate of catastrophe is measured at cell edges thereby constraining the final CMT alignment^36^. Cell geometry is thus a contributor to CMT organization in plant cells.

However, computational modeling predicts that this bias may be weak. To test whether the microtubule alignment according to cell shape is robust, the growth direction of microtubules was biased in silico: growth occurs from the microtubule plus end with a small directional noise; when this noise was biased by a cue in a direction other than the longitudinal axis, with a weight of ~ 1% only, the final CMT orientation in virtual cells followed the direction of that bias^31^ (Fig. 2a). This study does not exclude a contribution of cell geometry in CMT orientation; in fact, it suggests that CMT orientation is determined by cell geometry by default (i.e., in the absence of another, prevalent cue). However, this model suggests that cell geometry is not a strong determinant of CMT orientation. This would be consistent with the observation that adjacent cells can exhibit consistent CMT co-alignment, despite having different shapes^5^; conversely, cell geometry would add noise to neighboring cells with different shapes if supracellular stress were not strongly anisotropic.

Interestingly, in the absence of strong supracellular cues, cell geometry would in fact be sufficient to bias the pattern of mechanical stress in the wall. Typically, for a single pressurized elongated cell, maximal tension is transverse to the long axis of the cell^37^ (Fig. 2c). Using finite element models, the subcellular pattern of stress in the outer walls of adjacent cells in the epidermis was calculated^38,39^: tension in the (outer) wall should be twice higher along the circumference than along the long axis of the cell whether the cell is isolated or in a tissue. Therefore, when including the mechanical implications of cell geometry, and assuming that CMTs would align with maximal tension, such a cue may in principle be enough to override the purely steric impact of local curvature and cell shape on the final CMT array alignment.

Box 2 Putative anchoring mechanisms for CMTs Whereas the extracellular matrix–plasma membrane–actin continuum is rather well described and understood in animal cells, the exact nature of the cell wall–plasma membrane–CMT continuum is largely unknown in plants. Several proteins connecting the plasma membrane and the cytoskeleton have been identified^92–94^ and the lateral movement of several plasma membrane proteins is constrained by their interaction with the cell wall^95^. Electron microscopy and total internal reflection fluorescence microscopy images clearly show that microtubules are anchored to the plasma membrane. Conversely, when a microtubule end detaches from the membrane, it becomes quite agitated owing to active cytoplasmic streaming underneath (see e.g.^96^). Physical links between the plasma membrane and the cell wall are easily visualized upon partial plasmolysis, forming the so-called “Hechtian strands”. Such anchoring points are usually associated with plasmodesmata. As their number is relatively low, and despite the high bending stiffness of microtubules, plasmodesmata anchoring points would not be sufficient to explain the attachment of all CMTs to the plasma membrane. A second, non-exclusive, mechanism involves proteins that bind both microtubules and phospholipids. For instance, phosphatidic acid can recruit MAP65, which binds and bundles microtubules^97^; PIP2 biosensors have also been reported to accumulate in mechanically stressed regions where CMTs are stably co-aligned^98^. Such interactions may provide a relatively direct membrane anchoring mechanism: the fluidity of the membrane would allow a degree of freedom in CMT reorientation, and the CMT self-organization together with the indirect connection of the CMT network to fixed points (plasmodesmata), could maintain a stable cell wall–plasma membrane–CMT continuum. Note here that, at the plasma membrane, several receptor-like kinases exhibit an Arg–Gly–Asp (RGD)-binding motif, which may bind wall components, in a way analogous to integrins in animal cells binding to fibronectin RGD motifs^99^. Consistent with this idea, the plasma membrane tends to detach from the cell wall upon treatment with free RGD peptides^100^. Last, as CMTs are indirectly bound to the cellulose synthase machinery through CSI^101^ and CMU^102^ proteins, CMTs may also be anchored to the membrane in part via the cellulose synthesis machinery

## Microtubule stabilization by tension in in vitro assays

**Fig. 3:**
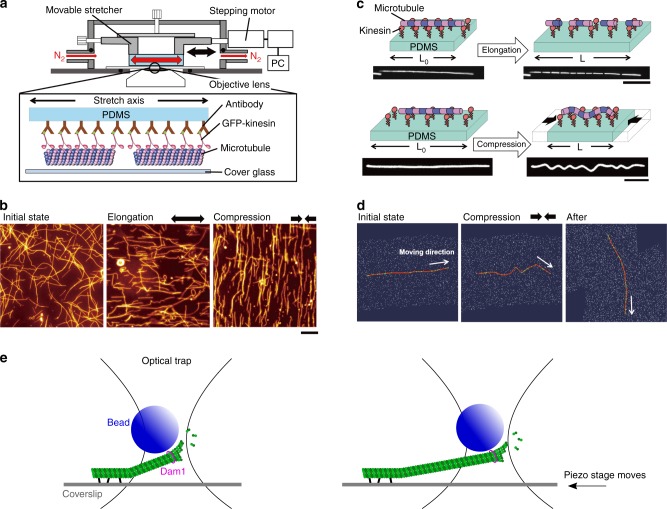
In vitro microtubules under mechanical stress. **a** Schematic diagram of an in vitro system to apply tension and compression to gliding microtubules on a kinesin-coated elastomer substrate (adapted from ref. ^43^). **b** Microtubules driven by surface immobilized kinesins align along maximal tension in vitro and conversely align against compression direction (adapted from ref. ^43^). **c** Fragmentation and buckling of microtubules at a stationary state induced by external tension and compression (adapted from ref. ^90^). **e** Using optical tweezer, growth of single microtubule is promoted when under tension along the direction of protofilaments (adapted from ref. ^41^, not to scale). **d** Microtubule aligns toward the direction that minimizes accumulated bending energy in silico (adapted from ref. ^43^)

Is there any evidence that microtubules can align with tension on their own? Several in vitro studies have addressed this issue. In gliding assays, where stable microtubules are propelled by surface-anchored motor proteins (kinesin and dynein), populations of microtubules move toward random directions on a planar surface, which mimics the displacement of CMTs. Recently, thanks to a coupling between stretchable polydimethylsiloxane (PDMS) substrate and the conventional gliding assay system, when microtubules gliding on a PDMS were subjected to tension by elongation of the substrate, the randomly moving microtubules aligned themselves along the tension lines. As the application of tensile stress was transient, orientation of microtubules became random soon after the release of tension. Conversely, microtubules that were put under compression in the same set-up re-aligned to be orthogonal to maximal compression direction (Fig. 3a, b). However, when stationary microtubules were subjected to tensile or compression stress, they underwent fragmentation and buckling, respectively^40^ (Fig. 3c). These results suggest that microtubules are able to reorganize when the stress pattern undergoes rapid changes and self-organize in aligned arrays in the direction of maximal stretch.

In polymerization assays of microtubules where microtubules growth is catalyzed by tiny stabilized microtubules (so-called seeds), the growing end of microtubules stretched by optical tweezers were found to prominently grow under tension^41,42^ (Fig. 3e). More specifically, beads coated with a kinetochore protein (Dam1), which stably binds microtubule ends, were attached at microtubule ends, trapped and then pulled using an optical tweezer. Microtubule shrinking rate was reduced to one third of its initial value (from 158 nm/s to 56 nm/s) when the applied tensile force was increased from 0.5 to 2 pN, showing that tension can slow down microtubule depolymerization. Note that when switching between different force regimes, with abrupt changes in force magnitude, microtubule shrinking rate was also immediately affected. This suggests that the effect of tension on microtubule is direct and that microtubules are able to perceive changes in force magnitude. Using a similar strategy, albeit using XMAP215-coated beads, the mean polymerization rate was twice higher when the pulling force was ~1 pN when compared with forces smaller than 0.5 pN. This demonstrates that tension promotes the polymerization activity of XMAP215 on microtubules. Interestingly, like XMPA215, both microtubule organizer1 (MOR1) and CLASP in plants contain plus end stabilizing TOG domains, and thus are in good positions to affect microtubule polymerization in a force-dependent way.

**Fig. 4:**
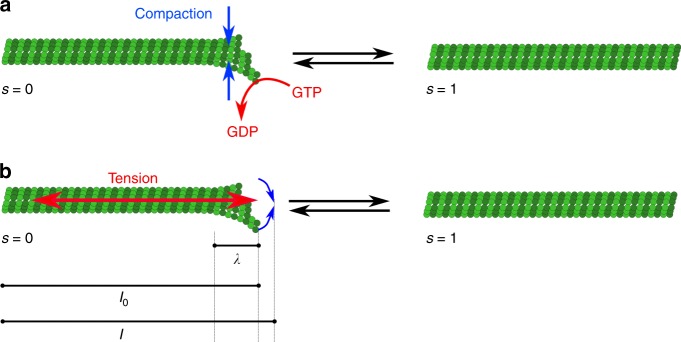
An energy-based mathematical model of tension sensing in a single microtubule. The model is based on a one-dimensional two-state mechanical model of tubulin protofilament alignment, through GTP hydrolysis **a** or external pulling force **b**, as illustrated. State variables are: the real-valued actual length $l \in {\Bbb R}$ of a stretchable segment of MT (e.g., anchor point to  plus end); a binary-valued indicator variable *s* ∈ {0,1} for the mechanical state of the lengthwise protofilaments at the  plus end cap (*s* = 0⇒splayed, *s* = 1⇒ aligned); and optionally a binary-valued indicator variable *σ* ∈ {0,1} for internal biochemical sensing of the mechanical state *s*. Principal exogenous parameters are $\lambda \in {\Bbb R}^{ \ge 0}$, the length of the splayable subregion; $l_0 \in {\Bbb R}^{ \ge \lambda }$, the segment resting length when aligned (so *l*_0_–*λ* is the resting length when splayed); *τ* = externally applied tension; *μ*_*s*_ = energy bias in favor of (or, if negative, against) alignment *s* = 1; *μ*_*σ*_= energy bias in favor of *σ* = 1; *α* = energetic reward for agreement of *s* = 1 and *σ* = 1. Given this notation, a Hooke’s law mechanical spring energy with two states can be written as: *E*_*mech*_ = (*k*/2)[*s*(*l*–*l*_0_)^2^+(1–*s*)(*l*–(*l*_0_–*λ*))^2^]–*τ*(*l*–(*l*_0_–*λ*)). Additional energy terms specific to discrete end cap state and sensing are: $E_{discrete} = - \mu _ss - \mu _\sigma \sigma - \alpha s\sigma$; then the total energy is *E*(*l*,*s*,*σ*) = *E*_*mech*_+*E*_*discrete*_. State probability follows the Boltzmann distribution, exp(–*βE*)/*Z*(*β*,params) where *Z* normalizes the distribution. Even ignoring *σ* (case *α* small) one obtains a double-well potential in the free energy *F*(*τ*) = −(1/*β*)log*Z* with two minima as a function of tension, one of them near *τ* = 0. This indicates that nonzero tension can be stabilized by the *s* = 1 mechanical protofilament alignment state which is in turn correlated (for *α* ≠ 0) with *σ* = 1 tension sensing. The readout state *σ* = 1 could in turn be amplified biochemically by, e.g., a phosphorylation/dephosphorylation cycle as in ref. ^91^, assuming that *σ* affects such enzymatic activity

Altogether, these in vitro studies suggest that the tension experienced by a microtubule can have a determining effect on the microtubule spontaneous behavior. How stress is sensed is a question that must be addressed both by computational scientists and structural biologists. For instance, an in silico study disclosed that microtubule orientation is biased by applied mechanical stimuli in order to minimize the accumulated bending energy in the microtubule shaft under compression of the substrate^43^ (Fig. 3d). Here we formulate a simple energy-based mathematical model of tension sensing in a single microtubule end, based on a one-dimensional two-state mechanical model of tubulin protofilament alignment as illustrated in Fig. 4. A macroscopic mechanical analogy for this model could be made to the two states of a hemispherical deflated ball: side A outside and side B inside (state *s* = 0), or vice versa (state *s* = 1). Parameter *μ*_*s*_ represents the difference in mechanical energies between the two states, and controls which state (if either) has the lower energy and the higher probability. Note that energy differences at this molecular scale would be comparable to thermal energy fluctuations. Using such a model one can evaluate the free energy associated with each value of an externally imposed tension *τ*. The result is a double-well potential, with one local minimum energy near *τ* = 0 corresponding to the splayed state of microtubule protofilament sheets and another local minimum for larger *τ* corresponding to the aligned state of microtubule protofilaments, whether the input is biochemical (GTP hydrolysis) or mechanical (imposed tension). In this way, external tension could indeed stabilize the aligned state of the MT end cap. At the molecular level, one may check whether the aligned state promotes the recruitment or activity of XMAP215, which would then catalyze tubulin subunit incorporation.

Recent progress in cryoEM may help us relate microtubule dynamic instability and mechanical stress within the lattice^44^. For instance, high-resolution images reveal that GTP hydrolysis changes the conformation of α-tubulin, leading to tubulin dimer compaction along the axis of protofilaments, and thus generating tension in the lattice^45,46^. Although it remains to be explained how external tension can interfere with this structural response, microtubule stability may very well depend on their tensile status. In other words, we are now closer to causally linking the intrinsic structure of the microtubule to its ability to withstand tension, while being destabilized by compression. This mechanical asymmetry, together with their elongated, anisotropic, shape, could be sufficient to make them tension sensors on their own.

## Cell wall contribution to the CMT response to stress

Based on the in vitro experiments discussed above, microtubules are stabilized by tension. In the simplest in vivo scenario, changes in external tension from the cell wall would be transferred to microtubules. This is by far the strongest assumption of this article, for at least two reasons. First, the wall–membrane–microtubule continuum is ill-described (Box 2). Second, the tension-induced fragmentation of immobilized microtubules on stretched PDMS^40^ appears to be incompatible with the idea that wall tension stabilizes cortical microtubules in plant cells. At this stage, we can assume that the wall–membrane–microtubule continuum allows a certain degree of freedom for CMTs to keep some motility. Consistent with this assumption, electron microscopy data show that cortical microtubules in leaf epidermal cells can detach from the plasma membrane and, in such situation, they align with the longitudinal axis of the cell, which fits both constraints imposed by cell geometry (see Fig. 2) and the main shear stress imposed by cytoplasmic streaming. Interestingly, such behavior happens when cells have ceased to elongate, or when cells are treated with 1-butanol, which likely affects the microtubule anchoring to the plasma membrane^47^.

Before testing the hypothesis that microtubules can respond to changes in tensile stress direction in a real plant cell, experiments in wall-less protoplasts may provide some interesting indications. In particular, when protoplasts are stretched by centrifugation, CMTs align with the direction of maximal tensile stress^48^. However, such experiments may not be conclusive enough for our purpose, as they do not clearly distinguish the impact of cell geometry, cell strain, or stress. For instance, one could imagine that CMTs acquire their default organization along the new longitudinal axis of the protoplast, or that microtubules become parallel to maximal strain, rather than maximal tensile stress. Interestingly, in animal cells, non-spindle microtubules can also respond to similar deformations: they notably populate the leading edge of experimentally stretched fibroblasts, aligning with the directional of maximal stretch^49^, consistent with the CMT orientation in plant protoplasts stretched by centrifugation. However, it again remains difficult to distinguish the microtubule response to stress from other cues, such as strain or geometry. These examples highlight the need to clearly differentiate the putative contributions of stress and strain to microtubule dynamic behavior. Plant cells may offer a way to do this.

In a plant cell, the maximal direction of (plastic) strain (i.e. growth) is often perpendicular to the predicted direction of maximal tensile stress, because of the anisotropic properties of the cell wall. Indeed, cortical microtubules generally guide the trajectory of cellulose synthase complexes at the plasma membrane^17,50^. This implies that when microtubules align with tension, they also indirectly resist tension, through the synthesis of cellulose microfibrils in the maximal direction of tensile stress in the wall^5,14^. In the vast majority of cells, the tensile stress patterns are anisotropic. If CMTs align along maximal tensile stress directions, then the anisotropic reinforcement of the wall through the deposition of cellulose microfibrils would reduce stress in that direction during growth, possibly until stress in the formerly minimal direction becomes higher and CMT orientations are randomized or switch to the next maximal stress direction. Altogether, this means that the relation between microtubules, strain and tensile stress is more complex in plants, as plant cells tend to grow in a direction that is orthogonal to maximal tension. Consequently, in contrast to protoplasts, walled plant cells offer the unique opportunity to discriminate between the microtubule response to strain or stress.

## CMTs align with maximal tensile stress in plant tissues

**Fig. 5:**
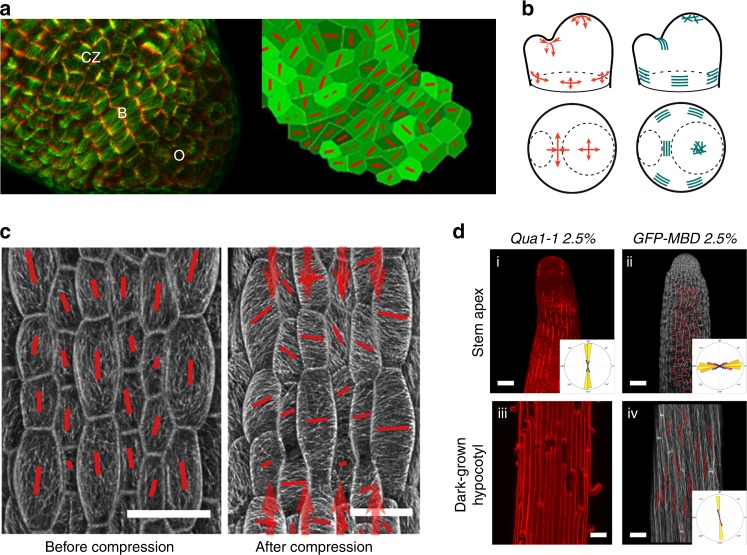
CMTs align along maximal tensile stress in plants. **a** Left: pattern of cortical microtubules at the shoot apical meristem (CZ: central zone, B: organ–meristem boundary, O: organ). Cell contours (red) and microtubules (green). Right: finite element model where local pattern of stress is predicted, with an emerging co-alignment of tensile stress directions (red bars) at the organ–meristem boundary domain (adapted from ref. ^5^). **b** Predicted pattern of mechanical stress at the shoot apical meristem (using a continuous model based on pressure vessel analogy), and matching supracellular microtubule pattern (adapted from ref. ^5^). **c** Pattern of cortical microtubules in light-grown hypocotyls before (left) and after (right) controlled compression along the axis of the hypocotyl (adapted from ref. ^52^). **d** Correlation between tension pattern derived from adhesion defects (bright propidium staining and cracks) in the *qua1* mutant in stems and basal region of dark-grown hypocotyls (left) and cortical microtubule orientation in a wild-type background (right). Microtubules are revealed by a GFP-Microtubule Binding Domain fusion (GFP-MBD) (adapted from ref. ^53^)

CMTs are usually perpendicular to the maximal growth direction (maximal strain) and they usually align parallel to predicted maximal tensile stress direction in plants. This has been repeatedly observed by different teams^5,15,51,52^, in different tissues (protoplasts^48^, epidermal peels^15^, hypocotyls^52,53^, shoot meristems^5^, cotyledons^38^, leaves^51^, immature seeds^54^, stems^53^, sepals^8^), at different scales, from subcellular^38^ to multicellular^5^, and using different micromechanical tests (stretching^15,52^, compression^5,51,55^, ablation^5^, drugs^30^) (Fig. 5). Note that the only cases where CMT orientation is not consistent with tensile stress pattern are in asymmetrically dividing cells (where an arc-shaped microtubule structure, the preprophase band, marks the next division site) and arguably in young hypocotyls, which exhibit constant rotations of their CMTs^56^.

In the following, we focus on cells at the shoot apical meristems where microtubule behavior has been analyzed, mechanical stress pattern has been modeled and several types of micromechanical perturbations have been applied. This tissue also offers a wide range of cell behavior, cells in the central zone growing slowly and isotropically, cells in the peripheral zone growing fast and anisotropically, and cells in the boundary domain growing slowly, being compressed between the organ and the meristem. Meristem cells in Arabidopsis resemble 5 × 5 × 5 μm cubes (±2 μm in the periclinal plane) with an outer cell wall that is about three times thicker than internal walls (300 nm vs. 100 nm). The epidermis is under tension and through indentation experiments, the meristem could be compared with a pressure vessel inflated by a pressure of ~1 MPa^57^.

Whereas meristematic cells are roughly isodiametric, CMTs are usually transverse in the peripheral zone and longitudinal in the organ–meristem boundary^5,58^ (Fig. 5a), further illustrating that cell geometry is not the sole prescriptor of CMT orientation. Similarly, CMTs are perpendicular to maximal strain direction in the peripheral zone, and parallel to maximal strain direction in the organ–meristem boundary^58^. Maximal strain is thus also unlikely to be a good prescriptor for CMT orientation. In fact, meristematic cell areal growth rate is ~2% per hour on average^30^, which, for a 5 μm wide meristematic cell, roughly corresponds to an elongation of 0.4 nm per minute, i.e., five orders of magnitude lower than microtubule growth rate. So far, the only cue that matches CMT orientation in the epidermis of the entire shoot apical meristem is maximal tensile stress: when the stress pattern at the shoot apical meristem is modified either by ablations, compressions or pharmacological treatments, CMTs change their orientation and, within 2 h, follow the new maximal tensile stress direction^5^ (Fig. 5b). Interestingly, the CMT response to stress at the shoot apical meristem was also shown to be independent of auxin^59^ and calcium^60^, thus further supporting the hypothesis that the CMT response to stress, at least in this tissue, may be more direct. If these experiments support the idea that CMTs are able to sense changes in stress direction, they do not necessarily imply that CMTs are also able to sense the stress pattern at steady state. In fact, based on these experiments and the in vitro results, CMTs may primarily sense changes in tensile stress direction.

A shortcoming in all above-mentioned experiments is that the stress pattern is always indirectly inferred: forces are invisible in essence, and cannot be visualized experimentally. Furthermore, most computational and mathematical models of stress are continuous and they focus on the epidermis, which is thought to be the load-bearing layer in most aerial plant organs. Typically, in a pressurized cylinder, maximal tensile stress is twice higher along the circumference, and such stress patterns may apply to stems or petioles. This means that such predictions usually do not take into account the contribution of internal tissues, nor do they consider the small heterogeneities and discontinuities that may alter the local stress pattern. Although these questions remain valid, predicted stress patterns in the epidermis have been indirectly validated by experiments. The load-bearing nature of the epidermis in aerial plant organs was notably revealed by performing cuts, the gaping pattern revealing the presence of tension in that outer layer^61,62^. The primary role of the epidermis in aerial morphogenesis was also further consolidated through molecular genetics experiments in which the whole phenotype of mutant plants can be rescued by expressing the wild-type gene in the epidermis specifically^63,64^. More recently, the tensile stress pattern in several plant organs was revealed, taking advantage of the *quasimodo* mutants, which exhibit cell–cell adhesion defects^53^. By using suboptimal osmotic conditions, such defects could be partially restored, and the direction of the resulting cracks could then be used to derive the anisotropy of tension in hypocotyls, stems, and leaves. Not only this study validated several predicted stress patterns, but it also revealed that CMTs usually align with maximal tensile stress in organs from wild-type plants grown in the very same conditions^53^ (Fig. 5d). Altogether, these results are consistent with a scenario in which CMTs are able to sense tension, and based on in vitro experiments, they may not require additional factors: they could spontaneously align with maximal tensile stress direction.

## A black box: how is stress in the wall transferred to CMTs?

Assuming a physical coupling between the cell wall and the microtubules (Box 2), changes in mechanical stress from the wall could in principle be transferred to microtubules. This is not straightforward. Most plant cells grow perpendicular to the most recent layer of cellulose microfibrils, meaning that if CMTs are aligned with maximal tensile stress, they are also aligned perpendicular to maximal strain. How can CMTs discriminate between stress and strain for their alignment? This is by far the most difficult question to address here. In the context of this perspective article, we provide below some speculations, and experimental suggestions. One of the main drawbacks here is that the exact relation between wall assembly and wall extension remains largely unknown^65^, with the possible exception of tip-growing cells like pollen tubes^66^.

**Fig. 6:**
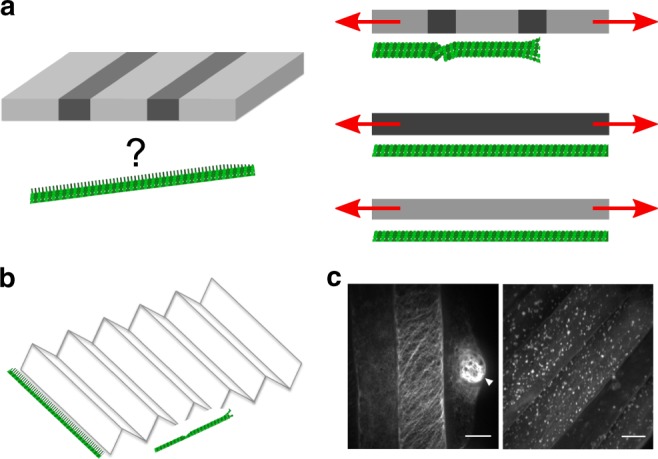
A role of wall heterogeneities to explain how microtubules distinguish maximal strain from maximal tensile stress. **a** Wall heterogeneities may induce strain discontinuities, destabilizing microtubules, whereas wall homogeneities (e.g., along or between cellulose microfibrils) may stabilize microtubules. **b** Assuming that wall heterogeneities would affect the roughness of the inner face of the wall, the smoother/straighter part of the wall may be parallel to maximal tensile stress direction, along which microtubules (green) would align. **c** Wall heterogeneity may arise from mechanical differences between cellulose microfibrils and the matrix; the delivery of component of the matrix is also heterogeneous in space and time, as shown by click chemistry with alkynylated fucose analogs in roots (left: late differentiation zone, right: early differentiation zone; adapted from ref. ^67^)

Because CMTs are physically anchored to the plasma membrane, tension in the wall may propagate to the CMTs, only if such tension was borne by homogeneous material in the wall. However, cell walls are mechanically and chemically heterogeneous. Such heterogeneities are likely to be actively maintained during wall synthesis and remodeling. For instance, the addition of matrix material through secretion (see e.g., ^67^ Fig. 6c) would allow strain to occur in any direction in principle, but the mechanical anisotropy of cellulose microfibrils biases this effect, by constricting growth direction and only allowing wall deformation between microfibrils. Similarly, the wall remodeler expansins do not promote cell growth through microfibril hydrolysis, but are thought instead to promote polymer creep and increase the spacing between microfibrils^68,69^. This provides a picture of the wall with aligned cellulose microfibrils where tensile stress is high and directional on the one hand, and domains where matrix material accumulates and for which the mechanical status is much more uncertain on the other hand (Fig. 6a). Such mechanical heterogeneities in the wall could generate mechanical conflicts in CMTs.

In that scenario, CMTs may align along stretches of cell walls that are rather homogeneous mechanically, i.e., along cellulose microfibrils or between cellulose microfibrils, but not across alternating cellulose microfibrils and matrix domains (Fig. 6a). This could in principle be tested in in vitro gliding assays but would require building a heterogeneous and stretchable PDMS, which may be difficult to accomplish. Azobenzene lipid could be a good alternative, as corresponding membrane domains can be put under tension upon light stimulation^70^. The analysis of CMTs and cellulose microfibrils in the *mor1* mutant may also be revisited, as this mutant was successfully used in the past to uncouple the deposition of new cellulose microfibrils from pre-existing ones^71^.

The roughness of the cell wall could also contribute to the nexus between CMT orientation and tensile stress. The inner side of the cell wall is likely to be slightly ruffled, at least at the smallest scales, owing to the heterogeneity of wall components. As the plasma membrane is pushed against the wall in turgid cells, such bumps and valleys may affect the direction of CMT polymerization, (Fig. 6b). Yet, in turgid cells, it is unclear why small ruffles would be aligned with maximal tensile stress. One way to address that question might be to analyze wall shape in the presence of more or less tension. Interestingly, large wall-buckling events can be induced upon strong plasmolysis, and this has even been used to reveal the presence of mechanical conflicts across the wall thickness^72^.

Another possible mechanism involves the generation of microcracks as the stress pattern changes. If such abrupt deformations are transferred to the microtubules, they might also destabilize or stabilize them depending on their orientation. Needless to say that the presence of microcracks, the mechanical heterogeneity and roughness of the wall could all contribute to the microtubule response to changes in tensile stress direction.

Last, in addition to wall heterogeneity and shape, it is also possible that the wall integrity pathway^73^ has an important role in the relation between CMT orientation and tensile stress in the wall. Interestingly, most sensors have been shown to interact with matrix components so far^74–76^. When cellulose synthesis is artificially inhibited, cell walls can become thicker, an excess of matrix components compensating for the reduction in cellulose microfibrils^77^. This provides a feedback loop for the perception of stress magnitude, not stress direction: wall sensors would trigger the synthesis and delivery of matrix components until they are not pulled by tension anymore. This may indirectly affect CMTs and their relation to stress direction in the wall. For instance, if matrix components are synthesized in excess relative to cellulose microfibrils, this may actively maintain the biochemical and mechanical heterogeneity of the wall. The wall would actively maintain the direction of tension along cellulose microfibrils, because the excess of matrix material would only resist stress magnitude, not stress direction.

These hypotheses are highly speculative and other scenarios could be investigated. Yet, understanding the mechanical and chemical heterogeneity of the wall will likely be instrumental to explain why microtubules in plant cells can change their orientation when maximal tensile stress direction is modified. This is the main missing link behind the hypothesis that microtubules would align along tension on their own in vivo too.

## Implications in physiology and development

Our hypothesis raises several questions. First, it is well established that CMTs reorient rapidly in response to many cues, including light^78^ or hormones^79^. Similarly, CMTs constantly change their orientations in light-grown hypocotyls^56^. How could this be compatible with a spontaneous CMT response to stress? Although our goal here is not to analyze all scenarios and cues (typically, complex biochemical gradients could also explain supracellular CMTs alignment and their rapid reorientation), the above-mentioned results are not incompatible with the notion that microtubules can function as tension sensors. Indeed, one could expect that blue light rapidly reduces turgor pressure (consistent with the observation that a switch from darkness to light also triggers an immediate reduction in growth rate), and thus tensile stress in the wall: microtubules would switch from their tension-derived orientation (transverse) to their default cell geometry derived orientation (longitudinal, as observed^31^) upon light exposure. Similarly, the constant reorientations of CMTs in light-grown hypocotyls, is not incompatible with the idea that multiple and weak cues (geometry and mechanics) are competing to align CMTs. In fact, it has been proposed that growth direction and local mechanical perturbations compete to orient CMTs in hypocotyls^32^.

**Fig. 7:**
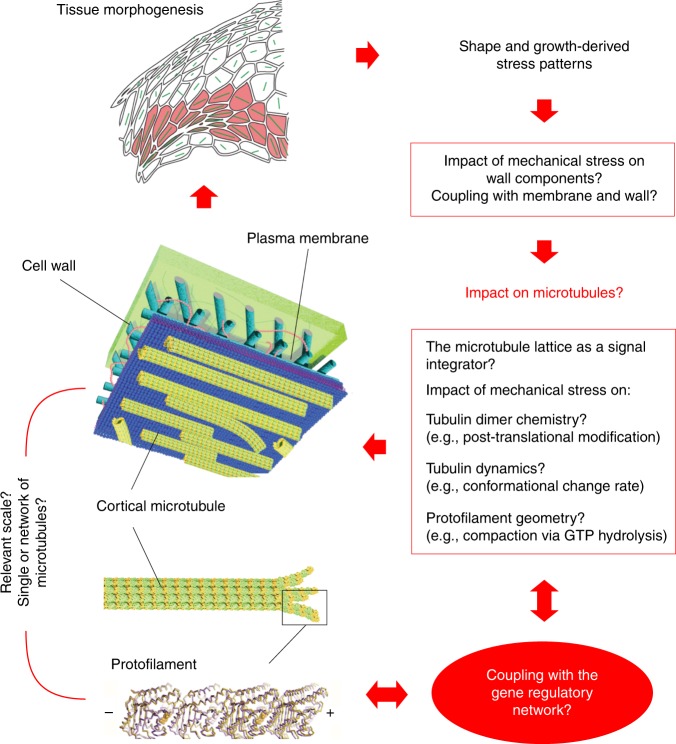
Integrating the microtubule-tension module in morphogenesis. Plant morphogenesis would emerge from the coupling between inputs from the gene regulatory network and an autonomous microtubule-tension amplifier. In that scenario, the microtubule lattice would be at the crossroad of the biochemical and mechanical control of growth. For instance, GTP hydrolysis within the protofilament leads to the compaction of the tubulin dimer (GTP in orange, GDP in pink—adapted from ref. ^46^) and this step may either be modulated by mechanical signals or mimicked by the impact of tension on the protofilament

Second, if our hypothesis is true, we arrive at a mechanical feedback, which requires very little molecular regulation: Upon a change in stress pattern, 1-tubulin dimer in a microtubule lattice would become more stable under tension, 2-Tensed individual CMTs would prevail, biasing the self-organization of CMT arrays in the cell, 3-CMT array alignment along maximal tension would in turn guide cellulose deposition to resist tension and channel the shape of most organs, 4-In turn, organ shape and growth would prescribe the tensile pattern and would maintain CMT orientations (Fig. 7).

Why would plants have selected such a simple mechanism, and what could be its evolutionary significance? Would not this quasi-autonomous mechanical feedback lock cell growth into a dead end, as room for regulation would be reduced to a minimum number of actors? The question of the why is beyond the scope of this article. Yet, it is tempting to propose that the presence of turgor pressure in the MPa range is a strong enough constraint for the cell to have an autonomous mechanism to resist it. If true, an obvious added value of such a self-sustaining CMT-based mechanical feedback would be to offer mechanical resistance by default, enabling fast-growing cell to constantly, rapidly and proportionally adjust to tensile stress in the wall. This could also explain why the relation between tension and microtubules is not as clear in animal cells, where osmotic pressure rather lies in the kPa range.

Last, even in a scenario where CMTs align along maximal tension on their own, the cell still hosts a wide array of potential regulators of the microtubule-tension feedback loop. For instance, the coupling between growth regulation at the cellular level and the microtubule-tension loop could involve modifications within the microtubule lattice. Local defects and post-translational modifications on tubulins could act as a code for molecular regulators to either enhance or reduce the microtubule response to tension (e.g., by modulating their dynamics, their ability to self-repair^28,80^, their anchoring to the membrane or their indirect interactions with cellulose microfibrils). For instance, the microtubule-severing enzyme katanin is preferentially recruited at lattice sites exhibiting defects^81^, and tubulin acetylation has been shown to mechanically stabilize microtubules^82,83^. The mechanical properties of microtubules are also dependent on bundling factors^84^, which likely modify their response to mechanical stress. Another point of coupling lies in the mechanotransduction pathways, which are rather adapted to sense stress intensity and also depend on biochemical signaling (channels, integrins, wall sensors). In fact, we propose here that wall sensors are blind to stress direction, and that this property may be important for CMTs to distinguish between stress and strain: by measuring an excess of tension in the wall, these sensors would promote the synthesis of material in the wall, resulting in a relative deficit of cellulose microfibrils and a relative excess of other components (pectin, hemicellulose), which would maintain a biochemical heterogeneity in the wall, possibly driving CMT orientation independent of cell strain (see Fig. 6).

If microtubules were sensors of tensile stress direction in plants, this would provide a parsimonious scenario in which the robustness of plant shapes would emerge from an autonomous response of microtubules to tension, and where hormones and other cues would regulate this central module either by affecting microtubule dynamics (e.g., with nucleating, bundling or severing factors, or with microtubule anchoring molecules), or by modulating tension levels (e.g., by stiffening or softening the cell walls). In turn, the microtubule response to tension would translate these cues in channeling growth direction, thus amplifying the effect of molecular triggers, locally, while not involving extra molecular control: the microtubule lattice, and its mechanical asymmetry, would be sufficient to provide a directional information for growth. This scenario corresponds to a division of labor between structure and architecture, as initial shape changes would primarily be orchestrated by the gene regulatory network, whereas implementation of shape changes would rely on an autonomous, self-organized, microtubule mechanical feedback (Fig. 7).

## Conclusion

Can the role of CMTs as tension sensors be extended to non-cortical microtubules? Kinetochores are interesting case studies for this question, because they couple chromosomes to microtubules, and their dynamics thus provide a force to allow chromosome segregation. The presence of bipolar kinetochores for instance is required for the proper segregation of chromosomes during mitosis (and meiosis II), and the opposing forces exerted by microtubules contribute to such polarity^85^. Conversely, would forces affect the coupling between microtubules and kinetochores? In an elegant experimental set-up, kinetochore–microtubule attachments were reconstituted using purified budding yeast kinetochores, and they were subjected to tensile stress (through cross-linking with beads and displacement with a laser trap). This showed that tension increases the lifetime of such kinetochore–microtubule attachments, notably by affecting several microtubule parameters such as polymerization rate, rescue rate, or catastrophe rate^86^. This is a typical case of catch-bond like association in which dissociation lifetime decreases when tension is applied. Interestingly, such phenomenon occurs at larger scales, such as in cell–cell adhesion: adhesion molecules adhere more tightly in the presence of tension^87^, and there is now increasing evidence that such adhesion molecules, like cadherins in animals or pectin in plants, are major players of mechanoperception pathways^88^.

Altogether these studies call for a deeper understanding of the links between microtubule biochemistry and mechanics^44^. Therefore, to conclude, here is a list of outstanding questions that remain to be addressed:

1. How does external tension affect the conformation of tubulin dimers and the properties of the microtubule ends and lattice?

2. How does bundling affect the microtubule response to mechanical stress?

3. What are the biochemical and mechanical features of the molecules coupling tension in the wall and cortical microtubules?

4. What are the implications of cell wall heterogeneity on stress propagation to the microtubules?

5. Where and what are the interplays between the molecular regulators of growth and the microtubule response to tension?

6. Does the heterogeneity and defects in the microtubule lattice act as a code for the microtubule response to tension, and thus for the regulation of plant cell growth?

7. What are the larger implications of microtubules aligning with tension, in development and beyond?
